# The Power of an Active Shooter Simulation: Changing Ethical Beliefs

**DOI:** 10.5811/westjem.2021.4.51185

**Published:** 2021-05-21

**Authors:** Maria-Pamela Janairo, Annemarie Marier Cardell, Michael Lamberta, Nubaha Elahi, Amish Aghera

**Affiliations:** * State University of New York Downstate Medical Center, Department of Emergency Medicine, Brooklyn, New York; † Kings County Hospital Center, Department of Emergency Medicine, Brooklyn, New York; ‡ Maimonides Medical Center, Department of Emergency Medicine, Brooklyn, New York; § Osceola Regional Medical Center, Department of Emergency Medicine, Kissimmee, Florida

## Abstract

**Introduction:**

During a hospital-based active shooter (AS) event, clinicians may be forced to choose between saving themselves or their patients. The Hartford Consensus survey of clinicians and the public demonstrated mixed feelings on the role of doctors and nurses in these situations. Our objective was to evaluate the effect of simulation on ethical dilemmas during a hospital-based AS simulation. The objective was to determine whether a hospital-based AS event simulation and debrief would impact the ethical beliefs of emergency physicians relating to personal duty and risk.

**Methods:**

Forty-eight emergency physicians and physicians-in-training participated in this cohort study based in an urban academic hospital. Simulation scenarios presented ethical dilemmas for participants (eg, they decided between running a code or hiding from a shooter). Surveys based upon the Hartford Consensus were completed before and after the simulation. Questions focused on preparedness and ethical duties of physicians to their patients during an AS incident. We evaluated differences using a chi-squared test.

**Results:**

Preparedness for an AS event significantly improved after the simulation (P = 0.0001). Pre-simulation, 56% of participants felt that doctors/nurses have a special duty like police to protect patients who cannot hide/run, and 20% reported that a provider should accept a very high/high level of personal risk to protect patients who cannot hide/run. This was similar to the findings of the Hartford Consensus. Interestingly, post-simulation, percentages decreased to 25% (P = 0.008) and 5% (P = 0.041), respectively.

**Conclusion:**

Simulation training influenced ethical beliefs relating to the duty of emergency physicians during a hospital-based AS incident. In addition to traditional learning objectives, ethics should be another important design consideration for planning future simulations in this domain.

## INTRODUCTION

An active shooter (AS), as defined by the Federal Bureau of Investigation (FBI), is one or more individuals actively engaged in killing or attempting to kill people in a populated area. Active shooter incidents have more than doubled between 2011–2018, with 27 reported in 2018.^1^,^2^,^3^ The Hartford Consensus was developed in an effort to address this growing issue, as well as to establish a national protocol to enhance survivability from AS and intentional mass casualty events by supporting the “run, hide, fight” algorithm to mitigate risk.^4^ Healthcare settings are uniquely vulnerable targets because patients may be unable to “run, hide, fight.” Making the decision to “run” creates an ethical dilemma for providers who have their own moral obligation not to abandon their patients. In a 2017 survey of the public and healthcare professionals, Jacobs and Burns found that both groups felt doctors and nurses had a special duty to protect patients similar to police officers and firefighters.^5^

Training healthcare providers how to respond to mass casualty incidents such as active shooters often involves active training exercises such as simulation. Outcomes of such training programs typically focus on improving knowledge and skills around the medical response to preserve life.^6^,^7^ The benefit of simulation-based training (SBT), as compared to didactic-based education, is that it allows the learner to have more time hands-on and encourages active participation. When studied side by side, simulation-based education was perceived as more enjoyable by students.^20^ and when teaching simulated patient emergencies, was found to generate superior team performance.^21^,^22^ Additionally, previous studies have used simulation to successfully evaluate resident response to ethical dilemmas.^23^

For this study, we were interested in using simulation to understand the physician perspective regarding personal duty and safety during an AS event. We hypothesized that the SBT would provide a realistic AS experience and change the perception of emergency physicians with regard to personal risk and duty. The primary study objective was to determine how the ethical beliefs of physician duty and personal risk are affected by a SBT exercise grounded in the “run, hide, fight” approach. Secondary objectives included the effect of SBT on their overall level of risk and preparedness for an AS event.

## METHODS

### Study Design

This was a cohort study to determine the perceptions of physicians regarding AS events before and after a SBT exercise. Survey questions and response options mirrored those used by the Hartford Consensus.^5^ The study was classified as “exempt” by the local institutional review board.

The SBT was an active, operations-based functional exercise in crisis management rather than a discussion-based approach. The goal of this approach was to create an experience to allow learners to reflect on their roles when confronted with an in-hospital AS. The operations-based format challenged participants to make quick decisions and to act definitively in their perceived roles during a crisis. Simulation scenarios were designed to replicate the tension that may occur for participants responding to an AS while actively engaged in patient care. During the post-simulation debriefing, facilitators reviewed the “run, hide, fight” protocol while encouraging learners to actively reflect on their beliefs regarding duty to patients and personal safety.

**Population Health Research Capsule**

What do we already know about this issue?

*During a hospital based active shooter (AS) event, clinicians may be forced to choose between saving themselves or their patients.*

What was the research question?

*Can simulation based training impact the ethical beliefs of physicians relating to personal duty and risk?*

What was the major finding of the study?

*Simulation training influenced ethical beliefs relating to the duty of physicians during an AS incident.*

How does this improve population health?

*In addition to traditional learning objectives, simulation can impact ethical beliefs and educators should consider this when developing curriculum.*

### Study Setting and Sample

The study was conducted at a private, urban hospital in the Northeast with an annual census of 120,000 patients, and associated Accreditation Council for Graduate Medical Education-accredited three-year emergency medicine (EM) residency and pediatric EM (PEM) fellowship programs. Participants consisted of a convenience sample of available EM attendings, EM residents, PEM fellows, and rotating fourth-year medical students who were available for Wednesday conference. We also chose to include available students as they actively contribute as care providers as part of the holistic team in our clinical setting. The SBT exercise was conducted during typical time reserved for education (Wednesday conference), which is generally mandated for all residents and fellows. Trainees were given the opportunity to opt out a day in advance through private correspondence over email, given the potential threat to psychological safety from an active shooter SBT.

### Measurements

Participants completed surveys immediately before and after the completion of the SBT exercise. Survey questions closely mirrored those previously used by the Hartford Consensus, with minor adaptations to collect basic data and to specifically reference the clinical environments staffed by physicians working at the local institution. Detailed demographic data regarding race, gender, and age were not included in the survey design due to concerns that with a small cohort of colleagues it would lead to identifiable responses. The final survey questions are presented in Table 1, and a copy of our final instrument as viewed by our respondents is in Appendix 1 as well as a copy of our survey results in Appendix 2.

Lastly, participants were sent a link to provide anonymous feedback on a rating scale from 1 to 5 on the actual SBT exercise related to the following: clarity of learning objectives; orientation to simulation environment; realism of simulation; relevance to practice; psychological safety; and effectiveness of debriefing. They were also afforded the opportunity to provide additional written feedback.

### Validity Evidence of Survey Tool

The survey was adapted from the Hartford Consensus study by Jacobs and Burns wherein these authors worked with an independent research firm specializing in probability-based survey research design. The survey questions were copied verbatim for our population, with the only change specifying the name of the hospital and other venues where the subjects worked. In the pre-briefing the authors instructed participants to respond based on their own personal beliefs as there may not be one “correct” answer to these questions. There were no consequences to our participants in relation to how they responded to survey questions with an opt-out option, which nobody chose. We did not measure the relationship of participant responses to other known variables as we were unaware of specific measures that would predictably relate to ethical beliefs.

### Simulation-based Training Design

Reference material on best practices managing AS events was sent to all potential participants one week prior to the SBT exercise.^8^,^9^ As part of standard curricular processes, trainees were assigned preparatory questions to answer in advance of the session to help prime them to successfully manage the event. Prior to the scenarios, participants underwent a pre-briefing that focused on their psychological safety and pushing their comfort levels, as well as addressing the basic assumptions in simulation.^10^ Participants were again given an opportunity to opt out of the scenario at any time before or during the scenario. No participants chose to opt out prior to or during the scenario.

Four scenarios were run simultaneously in adjacent mock clinical rooms within the Center for Clinical Simulation at the local institution. Each scenario was designed by experienced simulation faculty to present an ethical dilemma to the participants on whether they should independently “run, hide, fight” vs co-manage patients (Table 2). The scenarios were designed to specifically address an ethical dilemma complicating the participants’ abilities to run, hide or fight. A total of five trainees were present in each room, as well as two faculty members whose responsibility was to role-play within the scenario and to push the trainees to make difficult decisions while ensuring their psychological safety. Faculty members used their roles to prompt trainees to make difficult decisions regarding prioritizing patient care vs prioritizing personal safety as the simulation evolved. This is one of the benefits of SBT: Faculty can adjust the script in real time to engage quiet participants, foster debate, and encourage discussion about team priorities.

The simulation started with a recording of gunshots played from a portable speaker located in the hallway outside the respective scenario rooms. To generate ambiance during the scenario the portable speaker was moved up and down the hallway and periodic additional “gunshots” were fired. A group debriefing followed to address the various reactions that arose in response to various ethical dilemmas. This debriefing also emphasized the “run, hide, fight” algorithm and broke down scenarios specific to our ED and affiliated venues on where to hide or run if ever faced with this situation. The total length of the session was approximately 90 minutes and was repeated for a second group of learners.

### Data Analysis

Survey responses were presented using descriptive statistics. We evaluated differences in responses before and after the SBT using a chi-squared test. A *P* value of <0.05 was considered statistically significant. We used SPSS version 24 (IBM Corp., Armonk, NY) to analyze the data.

## RESULTS

Forty-eight emergency physicians and physicians-in-training participated in the SBT exercise (15 postgraduate year [PGY]1 EM residents, 7 PGY2 EM residents, 10 PGY3 EM residents, 5 PEM fellows, 8 EM attendings, and 3 medical students). Three EM faculty participants with prior knowledge of the Hartford Consensus survey and implicit knowledge of the study design were excluded from completing the survey as they would not be able to answer questions without inherent bias. Of the remaining 45 participants, 44 completed a pre-simulation survey (98% of participants) while 45 completed a post-simulation survey (100% participation). None of the participants chose to opt out of the simulation training because of a preexisting threat to psychological safety. Of the 45 participants, 27% had previously been a patient who stayed overnight in a hospital: 12% in the prior 12 months; 29% between 1–5 years in the past, and 59% over five years in the past.

A perceived high or very high risk of an AS did not significantly change after the SBT. The perceived level of preparedness and the importance of being prepared did significantly increase after the SBT. The level of importance to be prepared for an AS event was high before and after the SBT. Specific results are summarized in Table 3 and 4.

Participants feeling that doctors and nurses have a special duty like police officers and firefighters to protect patients who cannot get out of harm’s way from an AS significantly decreased from 60% to 25% (*P* = 0.008). Of those who answered that physicians/nurses have a special duty, 32% felt strongly prior to the simulation, while 11% expressed this after the simulation (*P* = 0.243).

The ethical belief relating to a high or very high level of personal risk that doctors and nurses should accept to protect patients who could not get out of harm’s way decreased significantly from 21% to 5% (*P* = 0.041). If participants themselves were patients who were unable to get out of harm’s way, 98% expressed no opinion in regard to expectations of doctors/nurses to get them out of harm’s way. After the simulation, 100% expressed no opinion on the survey (*P* = 0.309). Similarly, participants expressed no opinion (100%) regarding whether doctors or nurses should be required to save the lives of patients during a hospital-based AS event. After the simulation, the results remained unchanged (100%), where participants had no opinion.

Anonymous feedback on the SBT was provided by 31 participants (69% response rate) and is summarized in Table 5. Written feedback about realism ranged from “failed to make me feel truly threatened” to “it gave me anxiety and palpitations.”

## DISCUSSION

The perceived level of risk of an AS incident within a hospital setting compared to a more public setting (ie, concert hall, stadium, etc.) in our study was consistent with the FBI study.^1^,^2^ Public spaces were seen as a greater risk than hospital settings. The overwhelming majority believed in the importance of being prepared for such an event in a hospital or hospital-staffed setting. This again stresses the importance of keeping a safe environment for vulnerable populations in a hospital setting, and the need for formal, AS training exercises.

The Office of the Assistant Secretary for Preparedness and Response, part of the Department of Health and Human Services, produced a comprehensive report to guide planning for an AS event in healthcare settings.^11^,^12^ The report recommends mental rehearsal to work through various response options, which leads to better preparation. Simulation-based drills take this a step further, creating scenarios in which healthcare workers can work through ethical dilemmas and practice the “run, hide, fight” algorithm. Our results support the perception that preparedness does in fact improve after SBT. One prior study did demonstrate that knowledge around active shooters improved after training, albeit with a significantly more elaborate and time-intensive curricular design on a military base.^7^ While our study did not explicitly test knowledge gains, the curricular design was significantly more feasible and replicable for any hospital with modest space and equipment resources. In fact, written feedback about the realism of our relatively low-fidelity simulation suggests that it was more than adequate for some learners. A potentially more relevant next step in evaluating the impact of active drills would be to study actual performance during in situ drills after SBT.

The findings show that most participants, prior to this intervention, perceived a duty to protect their patients during an AS scenario and were willing to accept a high level of personal risk to do so. They also demonstrate that AS simulations are an effective way to challenge this perception, reducing its prevalence among participants. Interestingly, pre-survey responses in our cohort were similar to health professional responses to the Hartford Consensus survey. They found 62% believed they had a special duty to protect patients, and 27% felt they should accept a high or very high degree of risk to help patients unable to get out of harm’s way. Post-survey responses demonstrated a significantly decreased sense of duty after SBT. We suspect that this relates to the experiential nature of simulation to provoke physical and emotional responses.^13^,^14^ These responses serve as the basis for changing learner frames after simulation.^13^,^14^

The debriefing of this SBT was rather open ended and focused on the “run, hide, fight” paradigm. During the debriefing the participants were asked about familiarity with the Hartford Consensus, and while there was some basic knowledge of its existence no participant identified as having an understanding of the consensus results. During the reflective process, some participants remained quite adamant that they would not be able to live with themselves if they did not do their best to protect their patients, while others opined that it was necessary to survive to be able to help manage victims and future patients. Others still expressed that they would help as many patients as possible within the limits of their personal safety. Ultimately, the degree of personal risk that a physician/nurse accepts is a choice. The SBT seemed to give our participants an opportunity to make an informed decision that they could be comfortable with if they were to have the unfortunate experience of needing to deal with the ramifications of those decisions from an actual AS event.

Ethics has traditionally been inadequately addressed in medical education.^15^ Prior reviews of teaching and assessment of ethics in undergraduate medical education (UME) found that students, deans, and course directors wished for it to be better integrated with their coursework.^16^,^17^ A key feature of SBT is that it is experiential, which allows for theoretical aspects of ethics to become more concrete. As compared to SBT, traditional education using didactics is mostly a passive experience for the learner. Simulation allows for active engagement and has several features that make it well suited for AS training in ways that are not feasible with a traditional classroom format. Simulation allows for feedback grounded in individual and team performance.^24^ Furthermore, SBT is adaptable to the needs of the learner based on their performance.

Embedded facilitators within a scenario can interact with participants allowing for an experience that will address the learning objectives regardless of their baseline knowledge or their ability to interact within the simulation.^25^ Using a simulated context allows facilitators to leverage principles of adult learning theory grounded in the belief that education is learner-centric, in stark contrast to didactic-based education which is educator-centric.^26^ Also, the ability to fully control the environment is important as educators can titrate the appropriate level of “stress” for the learner without putting them in actual danger.^24^ It is because of these benefits that we chose to use simulation to address our educational goals. In our review of the literature regarding the education of ethics in UME, we found that educators should provide “a set of skills for ethical analysis and decision making.”^18^ The fact that beliefs were altered after SBT suggests that this was an effective method for discussing ethics while simultaneously providing a practical framework to apply lessons AS events, it may also be useful to study other paradigms when “run, hide, fight” may not be feasible. Inaba and colleagues proposed an alternative of “secure, preserve, fight.”^19^ Training to this mantra using simulation may also serve to further aid healthcare professionals’ ability to protect themselves while still satisfying their duty to the patient.

## LIMITATIONS

This study was based out of a single, urban, academic EM program focusing on physicians, and thus its generalizability may be limited. This population may not reflect that of other programs. As with all observational studies, there is potential for confounders not predicted or identified by the authors. Additionally, as a simulation-based exercise the experience is highly dependent on facilitator experience leading to questions of generalizability. While a growing body of evidence supports that skills learned in the simulation laboratory do translate to practice, it is difficult to predict how quickly skills or practices decay without additional primers. Given that EM providers in particular are placed in a unique social and clinical setting, they are more likely to be prone to workplace violence, which might further impact how they perceive their ethical responsibilities over time. This study did not follow participants longitudinally for the stability of the change in their ethical beliefs. Additionally, we were unable to determine whether there was any hidden facilitator bias during the debrief in shaping the impact of the SBT. Lastly, compared to many mass casualty simulations, this SBT was relatively low fidelity and resource intensive, which may have blunted its potential impact for those participants who had difficulty immersing themselves in the scenario.

## CONCLUSION

Active planning and training for an active shooter event is critical. During a hospital-based AS event, clinicians may be forced to choose between saving themselves or their patients. The study demonstrates that simulation training can influence ethical beliefs relating to the duty of doctors and nurses during a hospital-based AS incident. This underscores the power of simulation to significantly impact learners, including relatively low-resource designs such as ours. In addition to traditional learning objectives, ethics should be another important design consideration for planning future simulations in this domain.

## Supplementary Information





## Figures and Tables

**Table 1:** Survey questions and response options.

1. Identification	PGY1, PGY2, PGY3, Fellow, Attending, Medical Student
2. Current level of risk for an active shooter at the hospital	Very High, High, Moderate, Low, Very Low
3. Current level of risk for an active shooter event at a hospital staffed event (Barclays, MSG, music festival, etc.)	Very High, High, Moderate, Low, Very Low
4. Current level of preparedness for an active shooter event at the hospital	Very Prepared, Somewhat Prepared, Not so Prepared, Not at all Prepared
5. Current level of preparedness for an active shooter event at a hospital staffed event (Barclays, MSG, music festival, etc.)	Very Prepared, Somewhat Prepared, Not so Prepared, Not at all Prepared
6. What is the importance of being prepared for an active shooter event at the hospital?	Extremely Important, Very Important, Somewhat Important, Not so Important, Not at all Important
7. What is the importance of being prepared for an active shooter event at a hospital staffed event (Barclays, MSG, music festival, etc.)	Extremely Important, Very Important, Somewhat Important, Not so Important, Not at all Important
8. Do doctors and nurses have a special duty like police officers and firefighters to protect patients who cannot get out of harm’s way from an active shooter?	Special duty, Beyond their duty
9. If you answered special duty, how strongly do you feel?	Strongly, Somewhat Strongly
10. What is the level of personal risk doctors and nurses should accept to protect patients who cannot get out of harm’s way?	Very High Risk, High Risk, Moderate Risk, Low Risk, None
11. If you were a patient unable to get out of harm’s way, would you expect doctors and nurses to put themselves at risk to protect you?	Y, N
12. Should doctors and nurses be required to try to save the lives of patients in an active shooter attack or should this be a personal choice?	Required, Personal Choice
13. Have you been a patient in a hospital?	Y, N
14. How long ago was the last time you were a patient in a hospital?	Past 12 months, >1 year ago but <5 years ago, >5 years ago
15. Have you ever stayed overnight as a patient in a hospital?	Y, N

* All questions provided a “No Opinion” answer choice.

*PGY*, postgraduate year; *MSG*, Madison Square Garden; *Y*, Yes; *N*, No.

**Table 2:** Brief descriptions of simulation scenarios including primary patient diagnosis, role of embedded participants, resources needed, and pertinent ethical dilemma.

	Scenario description	Patient: primary diagnosis	Role of embedded participant(s)	Resources needed	Ethical dilemma
Case 1	Run a witnessed cardiac arrest with a reversible cause.	Hyperkalemia from acute onset renal failure	Nurse	High fidelity mannequin with operator. Embedded simulation participant to play role of nurse	How do you prioritize the needs of a patient that may be able to be saved under different circumstances?
Case 2	Manage a patient with an acute stroke eligible for thrombolysis with actively concerned family at the bedside.	Acute stroke	Family Member and Patient	Embedded simulation participants to play roles of patient and family member.	How to prioritize the needs of a non-ambulatory patient with a treatable condition?
Case 3	Manage an acute ST- elevation myocardial infarction (STEMI) requiring percutaneous angiography.	STEMI	Patient and Nurse	Embedded simulation participants to play roles of patient and nurse	How do you care for a patient with a treatable condition during an MCI?
Case 4	Manage a non-ambulatory patient with knee pain while a wounded physician attempts to run into the examination room.	Fractured knee and GSW complicated by PTX.	Patient and injured staff member.	Embedded simulation participants to play roles of patient and injured staff	How do you prioritize the needs of an injured colleague?

*MCI*, mass-casualty incident; *GSW*, gunshot wound; *PTX*, pneumothorax.

**Table 3:** Summary results by training year for key questions.

	PRE	PRE	POST	POST	% Change (post – pre)	
	% H & VH	% L & VL	% H & VH	% L & VL	% H & VH	% L & VL

What is the level of risk at Maimonides Hospital?	18%	48%	24%	36%	6%	−12%
PGY1	14%	57%	13%	33%	−1%	−24%
PGY2	11%	56%	14%	86%	3%	30%
PGY3	33%	33%	30%	20%	−3%	−13%
PEM fellow	0%	40%	40%	0%	40%	−40%
Med student	0%	100%	33%	67%	33%	−33%
Attending	40%	20%	40%	20%	0%	0%

	% H & VH	% L & VL	% H & VH	% L & VL	% H & VH	% L & VL

What is the current level of preparedness at Maimonides?	7%	23%	53%	9%	47%	−14%
PGY1	7%	43%	33%	27%	26%	−16%
PGY2	11%	0%	57%	0%	46%	0%
PGY3	11%	0%	80%	0%	69%	0%
PEM fellow	0%	20%	60%	0%	60%	−20%
Med student	0%	0%	67%	0%	67%	0%
Attending	0%	60%	40%	0%	40%	−60%

	% SD	% BD	% SD	% BD	% SD	% BD

Do doctors and nurses have a special duty like police officers to protect patients?	45%	36%	20%	60%	−25%	24%
PGY1	43%	36%	20%	53%	−23%	18%
PGY2	57%	43%	14%	86%	−43%	43%
PGY3	67%	33%	30%	70%	−37%	37%
PEM fellow	20%	20%	20%	20%	0%	0%
Med student	50%	0%	0%	67%	−50%	67%
Attending	20%	60%	20%	60%	0%	0%

	% H & VH	% L & VL	% H & VH	% L & VL	% H & VH	% L & VL

What is the level of personal risk doctors should accept to protect patients who can’t get out of harm’s way?	17%	33%	4%	53%	−12%	20%
PGY1	21%	36%	0%	53%	−21%	18%
PGY2	29%	14%	14%	43%	−14%	29%
PGY3	11%	44%	0%	60%	−11%	16%
PEM fellow	0%	60%	0%	80%	0%	20%
Med student	0%	0%	0%	33%	0%	33%
Attending	20%	20%	20%	40%	0%	20%

*VH*, very high; *H*, high; *M*, moderate; *L*, low; *VL*, very low; *SD*, special duty; *BD*, beyond their duty; *PGY*, postgraduate year; *PEM*, pediatric emergency medicine.

**Table 4:** Pre- and post-survey results: perceived risk by location, current level of preparedness by location, and the importance of each location being prepared for active shooter events.

Location (question)	Pre-survey	Post-survey	P-value
Hospital (high or very high risk)	9%	13%	0.490
Hospital-staffed Public Event (high or very high risk)	17%	28%	0.181
Hospital (very prepared or somewhat prepared)	7%	57%	0.0001
Hospital-staffed Public Event (very prepared or somewhat prepared)	23%	76%	0.0001
Hospital (extremely or very important to be prepared)	88%	89%	0.326
Hospital-staffed Public Event (extremely or very important to be prepared)	100%	96%	0.329

**Table 5:** Anonymous participant scenario feedback on a scale of 1 (poor) to 5 (excellent).

Question	Mean rating
Clearly conveyed simulation objectives?	4.8
Orientation to learning environment?	4.8
Relevance to clinical practice?	4.3
How safe did you feed during the scenario?	4.5
Was the realism sufficient for the exercise?	3.8
Quality of debriefing to promote a dialog that enhanced knowledge, reflection, and provide clear/constructive feedback?	4.8

1= No/Poor or Not at All; 5= Yes/Excellent, or Extremely.
