# Supplementary material for: The Power of an Active Shooter Simulation: Changing Ethical Beliefs
Source: West J Emerg Med. 2021 May 21;22(3):510–7. doi: 10.5811/westjem.2021.4.51185 (PMC8202989; doi:10.5811/westjem.2021.4.51185)
Supplement: Supplementary file 1 [file wjem-22-510-s001.docx]

| **Survey on Active Shooter** | | | | | | | | | | |
| --- | --- | --- | --- | --- | --- | --- | --- | --- | --- | --- |
| *Anonymous Survey* | | | | | | | | | | |
|  |  | | | | | | | | | |
| Identification… | | | | | | | | | | |
| ○ | | | ○ | | ○ | | ○ | | ○ | |
| PGY1 | | | PGY2 | | PGY3 | | Pem Fellow | | Attending | |
|  |  | | | | | | | | | |
| What is the level of risk for an active shooter event at Maimonides Hospital? | | | | | | | | | | |
| ○ | | ○ | | ○ | | ○ | | ○ | | ○ |
| Very High | | High | | Moderate | | Low | | Very low | | No Opinion |
|  |  | | | | | | | | | |
| What is the level of risk for an active shooter event at a Maimonides staffed event (Barclays, MSG, music festival, etc)? | | | | | | | | | | |
| ○ | | ○ | | ○ | | ○ | | ○ | | ○ |
| Very High | | High | | Moderate | | Low | | Very low | | No Opinion |
|  |  | | | | | | | | | |
| What is the current level of preparedness for an active shooter event at Maimonides Hospital? | | | | | | | | | | |
| ○ | | | ○ | | ○ | | ○ | | ○ | |
| Very  Prepared | | | Somewhat prepared | | Not so  prepared | | Not at all  prepared | | No Opinion | |
|  |  | | | | | | | | | |
| What is the current level of preparedness for an active shooter at Maimonides staffed event (Barclays, MSG, music festival, etc)? | | | | | | | | | | |
| ○ | | | ○ | | ○ | | ○ | | ○ | |
| Very  Prepared | | | Somewhat prepared | | Not so  prepared | | Not at all  prepared | | No Opinion | |
|  |  | | | | | | | | | |
| What is the importance of being prepared for an active shooter event at Maimonides hospital? | | | | | | | | | | |
| ○ | | ○ | | ○ | | ○ | | ○ | | ○ |
| Extremely important | | Very important | | Somewhat important | | Not so important | | Not at all important | | No Opinion |
|  |  | | | | | | | | | |
| What is the importance of being prepared for an active shooter at Maimonides staffed event (Barclays, MSG, music festival, etc)? | | | | | | | | | | |
| ○ | | ○ | | ○ | | ○ | | ○ | | ○ |
| Extremely important | | Very  important | | Somewhat important | | Not so important | | Not at all important | | No Opinion |
|  |  | | | | | | | | | |
| Do doctors and nurses have a special duty like police officers and firefighters to protect patients who cannot get out of harm’s way from an active shooter | | | | | | | | | | |
| ○ | | | | ○ | | | | ○ | | |
| Special duty | | | | Beyond their duty | | | | No Opinion | | |
|  |  | | | | | | | | | |
| If you answered special duty, how strongly do you feel? | | | | | | | | | | |
| ○ | | | | ○ | | | | ○ | | |
| Strongly | | | | Somewhat strongly | | | | No Opinion | | |
|  |  | | | | | | | | | |
| What is the level of personal risk doctors and nurses should accept to protect patients who cannot get out of harm’s way? | | | | | | | | | | |
| ○ | | ○ | | ○ | | ○ | | ○ | | ○ |
| Very High Risk | | High Risk | | Moderate Risk | | Low Risk | | None | | No Opinion |
|  |  | | | | | | | | | |
| If you were a patient unable to get out of harm’s way would you expect doctors and nurses to put themselves at risk to protect you? | | | | | | | | | | |
| ○ | | | | ○ | | | | ○ | | |
| Yes | | | | No | | | | No Opinion | | |
|  |  | | | | | | | | | |
| Should doctors and nurses be required to try to save the lives of patients in an active shooter attack or should this be a personal choice? | | | | | | | | | | |
| ○ | | | | ○ | | | | ○ | | |
| Required | | | | Personal Choice | | | | No Opinion | | |
|  |  | | | | | | | | | |
| Have you been a patient in a hospital? | | | | | | | | | | |
| ○ | | | | | | ○ | | | | |
| Yes | | | | | | No | | | | |
|  |  | | | | | | | | | |
| How long ago was the last time you were a patient in a hospital? | | | | | | | | | | |
| ○ | | | | ○ | | | | ○ | | |
| Past 12 months | | | | > 1 year ago, but < 5 years ago | | | | > 5 years ago, | | |
|  |  | | | | | | | | | |
| Have you ever stayed overnight as a patient in a hospital? | | | | | | | | | | |
| ○ | | | | | | ○ | | | | |
| Yes | | | | | | No | | | | |
