# Supplementary material for: The Power of an Active Shooter Simulation: Changing Ethical Beliefs
Source: West J Emerg Med. 2021 May 21;22(3):510–7. doi: 10.5811/westjem.2021.4.51185 (PMC8202989; doi:10.5811/westjem.2021.4.51185)
Supplement: Supplementary file 2 [file wjem-22-510-s002.pdf]

Results by Training Year from Pre and Post Simulation Surveys

		Pre-Simulation Survey						*POST-Simulation Survey*						*PRE*	*PRE*	*POST*	*POST*
		VH	H	M	L	VL	NoOp	VH	H	M	L	VL	NoOp	% H & VH	% L & VL	% H & VH	% L & VL
**Q2**	**What is the level of risk at Maimonides Hospital?**	**2**	**6**	**14**	**16**	**5**	**1**	**4**	**7**	**17**	**10**	**6**	**1**	**18%**	**48%**	**24%**	**36%**
	PGY1	0	2	3	5	3	1	0	2	7	3	2	1	14%	57%	13%	33%
	PGY2	0	1	3	4	1	0	0	1	0	4	2	0	11%	56%	14%	86%
	PGY3	1	2	3	3	0	0	1	2	5	1	1	0	33%	33%	30%	20%
	Pem Fellow	0	0	3	2	0	0	1	1	3	0	0	0	0%	40%	40%	0%
	Med Student	0	0	0	1	1	0	1	0	0	1	1	0	0%	100%	33%	67%
	Attending	1	1	2	1	0	0	1	1	2	1	0	0	40%	20%	40%	20%

		VH	*H*	*M*	*L*	*VL*	*NoOp*	*VH*	*H*	*M*	*L*	*VL*	*NoOp*	% H & VH	% L & VL	% H & VH	% L & VL
**Q3**	**What is the level of risk at a Maimonides Event?**	**4**	**11**	**19**	**6**	**3**	**1**	**6**	**18**	**14**	**4**	**2**	**1**	**34%**	**20%**	**53%**	**13%**
	PGY1	1	3	7	1	1	1	2	6	5	0	1	1	29%	14%	53%	7%
	PGY2	0	2	3	4	0	0	0	3	3	1	0	0	22%	44%	43%	14%
	PGY3	2	1	6	0	0	0	1	5	2	2	0	0	33%	0%	60%	20%
	Pem Fellow	0	4	0	0	1	0	1	3	1	0	0	0	80%	20%	80%	0%
	Med Student	0	0	0	1	1	0	1	0	0	1	1	0	0%	100%	33%	67%
	Attending	1	1	3	0	0	0	1	1	3	0	0	0	40%	0%	40%	0%

		VH	*H*	*M*	*L*	*VL*	*NoOp*	*VH*	*H*	*M*	*L*	*VL*	*NoOp*	% H & VH	% L & VL	% H & VH	% L & VL
**Q4**	**What is the current level of preparedness at Maimonides?**	**0**	**3**	**27**	**10**	**0**	**4**	**0**	**24**	**14**	**4**	**0**	**3**	**7%**	**23%**	**53%**	**9%**
	PGY1	0	1	6	6	0	1	0	5	5	4	0	1	7%	43%	33%	27%
	PGY2	0	1	7	0	0	1	0	4	3	0	0	0	11%	0%	57%	0%
	PGY3	0	1	8	0	0	0	0	8	2	0	0	0	11%	0%	80%	0%
	Pem Fellow	0	0	4	1	0	0	0	3	2	0	0	0	0%	20%	60%	0%
	Med Student	0	0	0	0	0	2	0	2	0	0	0	1	0%	0%	67%	0%
	Attending	0	0	2	3	0	0	0	2	2	0	0	1	0%	60%	40%	0%

		VH	*H*	*M*	*L*	*VL*	*NoOp*	*VH*	*H*	*M*	*L*	*VL*	*NoOp*	% H & VH	% L & VL	% H & VH	% L & VL
**Q5**	**What is the current level of preparedness at a Maimonides event?**	**2**	**8**	**23**	**4**	**0**	**6**	**2**	**29**	**10**	**0**	**0**	**4**	**23%**	**9%**	**69%**	**0%**
	PGY1	0	3	9	1	0	1	0	7	5	0	0	3	21%	7%	47%	0%
	PGY2	0	2	4	1	0	2	0	6	1	0	0	0	22%	11%	86%	0%
	PGY3	2	2	4	0	0	0	2	5	3	0	0	0	50%	0%	70%	0%
	Pem Fellow	0	1	3	1	0	0	0	4	1	0	0	0	20%	20%	80%	0%
	Med Student	0	0	0	0	0	2	0	2	0	0	0	1	0%	0%	67%	0%
	Attending	0	0	3	1	0	1	0	5	0	0	0	0	0%	20%	100%	0%

		VH	*H*	*M*	*L*	*VL*	*NoOp*	*VH*	*H*	*M*	*L*	*VL*	*NoOp*	% H & VH	% L & VL	% H & VH	% L & VL
**Q6**	**What is the importance of being prepared for a shooter at Maimonides ?**	**25**	**12**	**5**	**0**	**0**	**2**	**30**	**9**	**4**	**1**	**0**	**1**	**84%**	**0%**	**87%**	**2%**

	PGY1	8	4	1	0	0	1	9	2	2	1	0	1	86%	0%	73%	7%
	PGY2	3	4	2	0	0	0	4	3	0	0	0	0	78%	0%	100%	0%
	PGY3	4	3	1	0	0	1	7	2	1	0	0	0	78%	0%	90%	0%
	Pem Fellow	4	0	1	0	0	0	4	0	1	0	0	0	80%	0%	80%	0%
	Med Student	2	0	0	0	0	0	3	0	0	0	0	0	100%	0%	100%	0%
	Attending	4	1	0	0	0	0	3	2	0	0	0	0	100%	0%	100%	0%

		VH	*H*	*M*	*L*	*VL*	*NoOp*	*VH*	*H*	*M*	*L*	*VL*	*NoOp*	% H & VH	% L & VL	% H & VH	% L & VL
**Q7**	**What is the importance of being prepared for an active shooter at a Maimonides event Q7**	**33**	**10**	**0**	**0**	**0**	**1**	**34**	**8**	**2**	**0**	**0**	**1**	**98%**	**0%**	**93%**	**0%**
	PGY1	9	4	0	0	0	1	10	3	1	0	0	1	93%	0%	87%	0%
	PGY2	5	4	0	0	0	0	5	2	0	0	0	0	100%	0%	100%	0%
	PGY3	8	1	0	0	0	0	9	1	0	0	0	0	100%	0%	100%	0%
	Pem Fellow	5	0	0	0	0	0	4	0	1	0	0	0	100%	0%	80%	0%
	Med Student	2	0	0	0	0	0	3	0	0	0	0	0	100%	0%	100%	0%
	Attending	4	1	0	0	0	0	3	2	0	0	0	0	100%	0%	100%	0%

		SD	*BD*	*NoOp*	*SD*	*BD*	*NoOp*	% SD	% BD	% SD	% BD
**Q8A**	**Do Doctors and nurses have a special duty like police officers to protect patients?**	19	15	8	**9**	**27**	**9**	**45%**	**36%**	**20%**	**60%**
	PGY1	6	5	3	3	8	4	43%	36%	20%	53%
	PGY2	4	3	0	1	6	0	57%	43%	14%	86%
	PGY3	6	3	0	3	7	0	67%	33%	30%	70%
	Pem Fellow	1	1	3	1	1	3	20%	20%	20%	20%
	Med Student	1	0	1	0	2	1	50%	0%	0%	67%
	Attending	1	3	1	1	3	1	20%	60%	20%	60%

		Ss	*SS*	*NoOp*	*S*	*SS*	*NoOp*	% S	% SS	% S	% SS
**Q8B**	**If you answered special duty, How strongly do you feel?**	6	13	13	**1**	**8**	**11**	**19%**	**41%**	**5%**	**40%**
	PGY1	3	3	5	1	2	4	27%	27%	14%	29%
	PGY2	1	3	0	0	1	1	25%	75%	0%	50%
	PGY3	2	4	3	0	3	3	22%	44%	0%	50%
	Pem Fellow	0	1	3	0	1	2	0%	25%	0%	33%
	Med Student	0	1	1	0	0	1	0%	50%	0%	0%
	Attending	0	1	1	0	1	0	0%	50%	0%	100%

		VH	*H*	*M*	*L*	*VL*	*NoOp*	*VH*	*H*	*M*	*L*	*VL*	*NoOp*	% H & VH	% L & VL	% H & VH	% L & VL
**Q9**	**What is the level of personal risk doctors should accept to protect patients who cant get out of harms way?**	**4**	**3**	**13**	**12**	**2**	**8**	**2**	**0**	**14**	**17**	**7**	**5**	**17%**	**33%**	**4%**	**53%**
	PGY1	1	2	3	3	2	3	0	0	4	6	2	3	21%	36%	0%	53%
	PGY2	1	1	4	1	0	0	1	0	3	2	1	0	29%	14%	14%	43%
	PGY3	1	0	4	4	0	0	0	0	4	6	0	0	11%	44%	0%	60%

	Pem Fellow	0	0	1	3	0	1	0	0	1	1	3	0	0%	60%	0%	80%
	Med Student	0	0	0	0	0	2	0	0	1	0	1	1	0%	0%	0%	33%
	Attending	1	0	1	1	0	2	1	0	1	2	0	1	20%	20%	20%	40%

		Y	*N*	*NoOp*	*Y*	*N*	*NoOp*	% Y	% N	% Y	% N
**Q10**	**If You were a patient would you expect doctors and nurses to put themselves at risk?**	6	29	6	**5**	**35**	**5**	**15%**	**71%**	**11%**	**78%**
	PGY1	2	10	1	1	12	2	15%	77%	7%	80%
	PGY2	1	4	2	0	6	1	14%	57%	0%	86%
	PGY3	1	7	1	1	9	0	11%	78%	10%	90%
	Pem Fellow	1	4	0	1	3	1	20%	80%	20%	60%
	Med Student	0	0	2	0	2	1	0%	0%	0%	67%
	Attending	1	4	0	2	3	0	20%	80%	40%	60%

		R	*PC*	*NoOp*	*R*	*PC*	*NoOp*	%R	% PC	% R	% PC
**Q11**	**Should doctors and nurses be required to try to save the lives of a patients in an active shooter situation?**	**5**	**32**	**5**	**0**	**42**	**3**	**12%**	**76%**	**0%**	**93%**
	PGY1	3	10	1	0	13	2	21%	71%	0%	87%
	PGY2	0	7	0	0	7	0	0%	100%	0%	100%
	PGY3	0	9	0	0	10	0	0%	100%	0%	100%
	Pem Fellow	0	4	1	0	4	1	0%	80%	0%	80%
	Med Student	0	0	2	0	3	0	0%	0%	0%	100%
	Attending	2	2	1	0	5	0	40%	40%	0%	100%

		Y	*N*	*Y*	*N*	% Y	% N	% Y	% N
**Q12A**	**Have you been a patient in a hospital?**	**28**	**14**	**30**	**15**	**67%**	**33%**	**67%**	**33%**
	PGY1	12	2	13	2	86%	14%	87%	13%
	PGY2	5	2	5	2	71%	29%	71%	29%
	PGY3	6	3	6	4	67%	33%	60%	40%
	Pem Fellow	1	4	1	4	20%	80%	20%	80%
	Med Student	1	1	2	1	50%	50%	67%	33%
	Attending	3	2	3	2	60%	40%	60%	40%

		< 12	*1-5*	*> 5*	*< 12*	*1-5*	*> 5*	% < 5	% > 5	% < 5	% > 5
**Q12B**	**How long ago was the last time you were a patient in a hospital?**	**4**	**9**	**21**	**4**	**10**	**20**	**38%**	**62%**	**41%**	**59%**
	PGY1	2	6	5	3	6	4	62%	38%	**69%**	**31%**
	PGY2	0	3	4	0	3	3	43%	57%	**50%**	**50%**
	PGY3	1	0	7	0	0	6	13%	88%	**0%**	**100%**
	Pem Fellow	0	0	1	0	1	2	0%	100%	**33%**	**67%**
	Med Student	0	0	2	0	0	3	0%	100%	**0%**	**100%**
	Attending										

Y	*N*	*Y*	*N*	% Y	% N	% Y	% N

**Q12C**	**Have you ever stayed overnight as a patient in a hospital?**	**13**	**29**	**12**	**33**	**31%**	**69%**	**27%**	**73%**
	How long ago was the last time you were a patient in a hospital?	0	0	0	0	0%	0%	0%	0%
	PGY1	4	10	4	11	29%	71%	27%	73%
	PGY2	2	5	2	5	29%	71%	29%	71%
	PGY3	4	5	3	7	44%	56%	30%	70%
	Pem Fellow	0	5	0	5	0%	100%	0%	100%
	Med Student	1	1	1	2	50%	50%	33%	67%

KEY:

VH	Very High	Y	Yes
H	High	N	No
M	Moderate	R	Required
L	Low	PC	Personal Choice
VL	Very Low	< 12	Less than 12 months
NoOp	No Opinion	1-5	Between 1 and 5 years ago
S	Strongly	>5	Greater than 5 years ago
SS	Somewhat Strongly		
